# Ouabain prevents pathological cardiac hypertrophy and heart failure through activation of phosphoinositide 3-kinase α in mouse

**DOI:** 10.1186/s13578-015-0053-7

**Published:** 2015-11-18

**Authors:** Jian Wu, Daxiang Li, Lingling Du, Mustafa Baldawi, Marjorie E. Gable, Amir Askari, Lijun Liu

**Affiliations:** Department of Biochemistry and Cancer Biology, College of Medicine and Life Sciences, University of Toledo, 3000 Arlington Ave., MS 1010, Toledo, OH 43614 USA; Center for Craniofacial Molecular Biology, University of Southern California, Los Angeles, CA USA; State Key Laboratory of Tea Plant Biology and Utilization, Anhui Agricultural University, Hefei, People’s Republic of China

**Keywords:** Na^+^/K^+^-ATPase, PI3 kinase, Cardiac hypertrophy, Heart failure, Ouabain, Digitalis

## Abstract

**Background:**

Use of low doses of digitalis to prevent the development of heart failure was advocated decades ago, but conflicting results of early animal studies dissuaded further research on this issue. Recent discoveries of digitalis effects on cell signal pathways prompted us to reexamine the possibility of this prophylactic action of digitalis. The specific aim of the present study was to determine if subinotropic doses of ouabain would prevent pressure overload-induced cardiac remodeling in the mouse by activating phosphoinositide 3-kinase α (PI3Kα).

**Results:**

Studies were done on an existing transgenic mouse deficient in cardiac PI3Kα (p85-KO) but with normal cardiac contractility, a control mouse (Con), and on cultured adult cardiomyocytes. In Con myocytes, but not in p85-KO myocytes, ouabain activated PI3Kα and Akt, and caused cell growth. This occurred at low ouabain concentrations that did not activate the EGFR-Src/Ras/Raf/ERK cascade. Con and p85-KO mice were subjected to transverse aortic constriction (TAC) for 8 weeks. A subinotropic dose of ouabain (50 µg/kg/day) was constantly administrated by osmotic mini-pumps for the first 4 weeks. All mice were monitored by echocardiography throughout. Ouabain early treatment attenuated TAC-induced cardiac hypertrophy and fibrosis, and improved cardiac function in TAC-operated Con mice but not in TAC-operated p85-KO mice. TAC downregulated α2-isoform of Na^+^/K^+^-ATPase but not its α1-isoform in Con hearts, and ouabain treatment prevented the downregulation of α2-isoform. TAC-induced reduction of α2-isoform did not occur in p85-KO hearts.

**Conclusions:**

Our results show that (a) safe doses of ouabain prevent or delay cardiac remodeling of pressure overloaded mouse heart; and (b) these prophylactic effects are due to ouabain binding to α2-isoform resulting in the selective activation of PI3Kα. Our findings also suggest that potential prophylactic use of digitalis for prevention of heart failure in man deserves serious consideration.

**Electronic supplementary material:**

The online version of this article (doi:10.1186/s13578-015-0053-7) contains supplementary material, which is available to authorized users.

## Background

In 1933, Christian [1] advocated the prophylactic use of digitalis to retard cardiac enlargement in patients with heart disease but without heart failure. In 1965, Williams and Braunwald [2] presented the first experimental support for this proposal showing that rats subjected to suprarenal aortic constriction and treated with daily nontoxic doses of digitoxin prior and following aortic constriction, exhibited less myocardial hypertrophy and lower incidence of fatal heart failure than rats subjected to aortic constriction but not treated with digitoxin. Though a number of studies that followed [3–5] either confirmed or questioned the above findings and conclusions, the issue of whether or not digitalis has a prophylactic effect on the growth of the overloaded hearts seems to have disappeared from the subsequent literature. Perhaps the question was raised ahead of its time. We have now reexamined the issue in the light of the recently appreciated effects of digitalis drugs on cell signaling pathways. Here, we present the results of initial studies using fresh experimental approaches to test for the possible prophylactic effect of digitalis on the heart that is subjected to pressure overload.

Digitalis drugs (such as digoxin, digitoxin, and ouabain) are highly specific inhibitors of the Na^+^/K^+^-ATPase of the plasma membrane of most of higher eukaryotic cells [6, 7]. This enzyme (the sodium pump) catalyzes the coupled active transport of Na^+^ and K^+^, maintains resting membrane potential, regulates cell volume, and enables the Na^+^-coupled transports of a multitude of nutrients and other ions across the cell membrane. Na^+^/K^+^-ATPase has two subunits (α and β) that are essential for ion pumping, and a third subunit (a FXYD protein) that regulates function [6, 7]. There are multiple isoforms of each subunit with tissue and species specificities, and variations among the digitalis sensitivities of the isoforms [6–8].

In the heart, digitalis concentrations that partially inhibit Na^+^/K^+^-ATPase to cause a modest increase in intracellular Na^+^ are sufficient to affect the robust Na^+^/Ca^2+^ exchanger of the myocyte plasma membrane, causing significant increases in intracellular Ca^2+^ and cardiac contractility [9, 10]. This positive inotropic effect has long been assumed by many to be the basis of the classical use of digitalis drugs for the treatment of heart failure [9, 10].

In more recent years, it has been realized that in addition to its vital ion transport function, Na^+^/K^+^-ATPase may also act as a signal transducer. Through digitalis-induced transient communications with neighboring membrane proteins, the digitalis-inhibited Na^+^/K^+^-ATPase activates multiple cell signaling pathways, leading to highly cell-specific downstream consequences [11]. In our early studies on the signaling function of the sodium pump in cardiac myocytes, we noted the apparent paradox that digitalis drugs which had long been used to treat the hypertrophied failing heart, caused hypertrophy of the cultured cardiac myocytes [12]. Subsequently, we found that this drug-induced hypertrophy is due to the activation of PI3K/Akt/mTOR pathway, and that digitalis treatment of the cultured myocytes activates PI3Kα but not PI3Kγ [13]. More recently, we also showed that ouabain-induced activation of PI3Kα and the resulting hypertrophy are independent of ouabain’s positive inotropic effect [14]. These findings, coupled with the extensive prior research of others [15] indicating the association of PI3Kα with physiological cardiac hypertrophy, and that of PI3Kγ with pathological hypertrophy, led us to suspect that digitalis-induced hypertrophy may indeed be akin to physiological hypertrophy [13, 14]. And since there is ample evidence to suggest that selective activation of PI3Kα not only induces physiological hypertrophy but may also prevent the detrimental effects of the stimuli that cause pathological hypertrophy [15], we hypothesized that the forgotten prophylactic effect of digitalis is due to the selective activation of cardiac PI3Kα. Here, we report the testing of this hypothesis in the mouse. We used ouabain as prototypic digitalis drug; transverse aortic constriction (TAC) to induce pressure-overload and pathological hypertrophy; and we compared the effects of ouabain treatment on the development and the consequences of TAC in the wild type mouse and a genetically modified mouse from which cardiac PI3Kα was deleted but exhibited normal cardiac contractility and histology [16]. Our findings supported the tested hypothesis.

## Results

### Comparison of heart size and function in normal mice and those deficient in PI3Kα

**Fig. 1:**
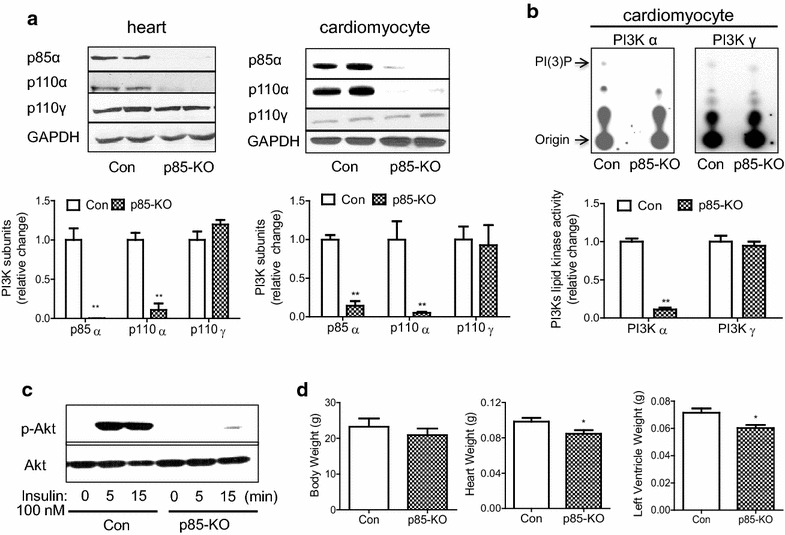
Characterization of p85-KO mouse hearts. **a**. Representative blots of p110α and p85 subunits of PI3Kα and p110 γ subunit of PI3Kγ in whole heart lysate (*top left*) and in freshly isolated adult mouse cardiomyocytes (*top right*). GAPDH was used as a loading control. Quantitative data of expression of PI3 K subunits in Con and p85-KO mouse hearts (n = 4) (*bottom left*) and cardiomyocytes (n = 7) (*bottom right*); **b** representative TLC plates show PI3Kα and PI3Kγ lipid kinase activities in freshly isolated Con or p85-KO cardiomyocytes (*top*). Quantitative data of PI3K activity in Con and p85-KO cardiomyocytes (n = 4); **c** insulin (100 nM)- induced Akt signaling in Con and p85-KO cardiomyocytes (representative blots of three experiments) and **d** comparison of body weight, heart weight and left ventricular weight in Con and p85-KO mice (*Con* n = 5, *p85-KO* n = 7). *P < 0.05, **P < 0.01 vs. Con

**Fig. 2:**
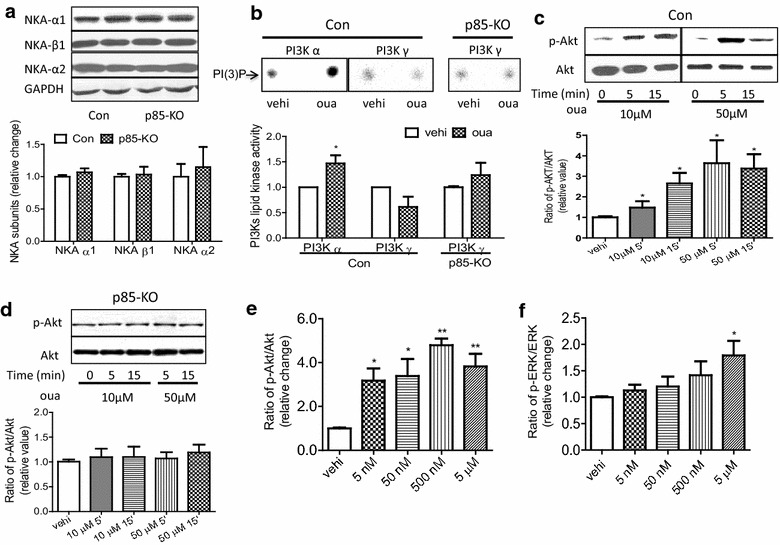
Comparison of ouabain-induced signaling in cultured cardiomyocytes from Con and p85-KO mice. **a**. Representative blots of Na^+^/K^+^-ATPase isoforms (*top*). GAPDH was used as a loading control. Quantitative data of expression of Na^+^/K^+^-ATPase isoforms (n = 6) (*bottom*); **b**. representative PI(3)P *dots* show the effect of ouabain (50 μM, 5 min) on PI3Kα and PI3Kγ lipid kinase activities in Con and p85-KO cardiomyocytes, vehicle (vehi) (*top*). Quantitative data of PI3K activity from the top. (n = 9, *P < 0.05 vs. vehi); **c** representative blots of the effect of ouabain on Akt activation in Con cardiomyocytes (*top*). Quantitative data of Akt activation from the *top* (n = 6, *P < 0.05 vs. vehi); **d** representative blots of the effect of ouabain on Akt activation in p85-KO cardiomyocytes (*top*). Quantitative data of Akt activation from the top. (n = 6, *P < 0.05 vs. vehi). **e** ouabain (5 min) dose- responses on Akt activation in wild-type cardiomyocytes (n = 6, *P < 0.05, **P < 0.01 vs. vehi) and **f** ouabain (5 min) dose- responses on ERK activation in wild-type cardiomyocytes (n = 6, *P < 0.05 vs. vehi)

In the present studies, which were designed to test our hypothesis on the prophylactic action of ouabain, we wished to use the mouse generated by Luo et al. [16] with the muscle specific deletion of p85α regulatory subunit and germ line deletion of p85β regulatory subunit of PI3Kα. Because this mouse has not been widely used since its generation, we deemed it necessary to confirm their main findings on the characteristics of this mouse. Our results summarized below are consistent with and reinforce those of Luo et al. [16]: (1) In the p85-KO mouse, relative to Cre mice (Control mice), protein levels of p85α and p110α of PI3Kα were greatly reduced in the lysates of the whole heart or the isolated cardiomyocytes, whereas the levels of p110γ of PI3Kγ remained unchanged (Fig. 1a); (2) in cardiomyocyte lysates of the p85-KO mouse, relative to those of the control, the basal level of PI3Kα activity was greatly reduced, while the activity of PI3Kγ was unchanged (Fig. 1b). The remaining 10 % of the PI3Kα activity in the lysate of the p85-KO myocytes is most likely due to myocytes that have escaped gene deletion as noted before [16], and as it has been established in cases of many other cardiac-specific deletions [14, 17]; (3) when intact cardiomyoyctes isolated from the p85-KO and the control mice were similarly exposed to insulin, the expected activation of Akt was noted in the control myocytes, but greatly reduced in the p85-KO myocytes (Fig. 1c). The serine/threonine kinase Akt, also known as protein kinase B (PKB), is a downstream component of the signaling cascades that are dependent on PI3 K activation at the insulin or other growth factor receptors [15, 16]; (4) The hearts of the p85-KO mice were smaller than those of control (Fig. 1d; Table 1), but cardiac contractility and function seemed to be normal in the KO hearts (Tables 1, 2).

**Table 1:** Echocardiographic analysis (baseline) of Con and p85-KO mouse hearts

	BW (g)	HR (bpm)	EDA (cm^2^)	ESA (cm^2^)	PWT (cm)	SWT (cm)	RWT	MPI	FS	LV mass (g)
Con	31.8 ± 1.2	439 ± 9	0.168 ± 0.004	0.088 ± 0.003	0.114 ± 0.003	0.104 ± 0.003	0.60 ± 0.02	0.36 ± 0.03	0.45 ± 0.02	0.126 ± 0.004
KO	28.4 ± 1.0	438 ± 11	0.153 ± 0.004*	0.081 ± 0.004	0.108 ± 0.005	0.087 ± 0.002*	0.54 ± 0.02	0.30 ± 0.02	0.45 ± 0.01	0.105 ± 0.005*

The p85-KO and the Cre controls (12–16 week-old, male) were subjected to echocardiography analysis

*BW* body weight; *HR* heart rate; *EDA* end diastolic area; *ESA* end systolic area; *PWT* posterior wall thickness; *SWT* septal wall thickness; *RWT* relative wall thickness; *MPI* myocardial performance index; *FS* fractioning shortening; LV *mass* left ventricular mass; *Con* n = 21; *KO* n = 15

* p < 0.05 *v.s.* Con

**Table 2:** Comparison of cardiac hemodynamic function (baseline) of Con and p85-KO mouse hearts

	BW (g)	HR (bpm)	Ves (LII)	Ved (LII)	Pes (mmHg)	Ped (mmHg)	SV (Lll)	EF (%)	dP/dt (mmHg/s)	−dP/dt (−mm Hg/s)
Con	23.1 ± 3.0	339 ± 32	14.8 ± 2.3	22.2 ± 1.2	79.2 ± 5.6	7.0 ± 2.7	10.2 ± 1.7	48.3 ± 8.4	4452 ± 924	3998 ± 858
KO	21.3 ± 1.8	271 ± 11	12.5 ± 2.0	17.8 ± 2.4	75.3 ± 1.8	6.5 ± 1.4	7.9 ± 1.1	44.4 ± 5.9	3732 ± 385	3092 ± 444

Hemodynamic function of p85-KO and the Cre controls (12 week-old, male) were measured by cardiac catheter as described in “Method”. There is no statistical significance between the Con and KO mice

*BW* body weight; *HR* heart rate; *Ves* end systolic volume; *Ved* end diastolic volume; *Pes* end systolic pressure; *Ped* end diastolic pressure; *SV* stroke volume; *EF* ejection fraction; *+dP/dt* the rate of pressure development; *−dP/dt* the rate of relaxation. *Con* n=4; *KO* n=7

### Comparison of Ouabain’s signaling effects on cardiomyocytes isolated from normal mice and those deficient in PI3Kα

Adult mouse cardiomyocytes are known to contain the α1 isoform, the α2 isoform, and the β1 isoform of the Na^+^/K^+^-ATPase [14, 18]. Since an αβ dimer is the functional unit to which ouabain binds [7], we immunoassayed the lysates of freshly isolated cardiomyocytes of the control and the p85-KO mice for α1, α2, and β1 protein, and found no significant differences between the isoform content in the two mice (Fig. 2a).

Our previous studies indicating ouabain-induced selective activation of cardiac PI3Kα but not PI3Kγ [13] were done on neonatal and adult cardiomyocytes of rat. To see if similar selective effects of ouabain are noted in adult mouse cardiomyocytes, intact myocytes isolated from control and p85-KO mouse hearts were exposed to ouabain for 5 min, and assayed for PI3Kα and PI3Kγ activity. In control myocytes, ouabain activated PI3Kα but not PI3Kγ activity (Fig. 2b). Ouabain also caused no significant activation of PI3Kγ of the p85-KO myocytes (Fig. 2b). Consistent with these findings, ouabain-induced activation of Akt during 5–15 min was observed in the control myocytes (Fig. 2c), but not in p85-KO myocytes (Fig. 2d). When further experiments were done to estimate the minimum dose of ouabain necessary for the rapid activation of Akt in control myocytes, ouabain concentration as low as 5 nM caused pronounced activation of Akt after 5 min treatment (Fig. 2e). Significantly, the activation of ERK 1/2 in the same control myocytes required much higher ouabain concentrations (Fig. 2f).

**Fig. 3:**
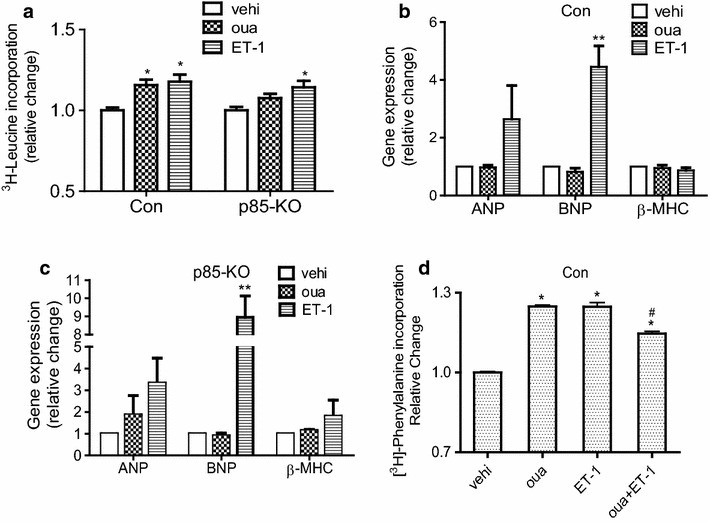
Effects of ouabain on hypertrophic growth and hypertrophic marker gene expressions in cultured cardiomyocytes of Con and p85-KO mice. **a** Comparison of 12 h treatment of ouabain (50 μM) and ET-1 (100 nM) on protein synthesis (^3^H-leucine incorporation) in Con and p85-KO cardiomyocytes (n = 6); **b**, **c** ouabain and ET-1 effects on hypertrophic markers (fetal gene expression) in Con and p85-KO cardiomyocytes (n = 3) and **d** effects of ouabain, ET-1 and the combination of the two on protein synthesis (^3^H-phenylalanine incorporation) (n = 9). *P < 0.05 vs. vehi, **P < 0.01 vs. vehi,^ #^P < 0.05 vs. oua

In isolated cardiomyocytes, hypertrophy of the terminally differentiated cells may be assessed by measuring the initial rate of protein synthesis [19, 20], and we have shown before that ouabain-induced activation of PI3Kα/Akt pathway leads to increase protein synthesis in isolated mouse adult cardiomyocytes [14]. Comparing protein synthesis in the control and the p85-KO myocytes, we found that ouabain (50 μM, 12 h) stimulated hypertrophy significantly in the former but not in the latter (Fig. 3a). Importantly, endothelin-1 (ET-1) which is known to cause pathological hypertrophy through the activation of PI3Kγ [15], increased protein synthesis in both the control and the p85-KO myocytes (Fig. 3a). Atrial natriuretic peptide (ANP), brain natriuretic peptide (BNP) and β-myosin heavy chain (β-MHC) are commonly used cardiac hypertrophic markers [15]. In agreement with our previous findings [14], and consistent with the present findings of Fig. 3a, ET-1 (100 nM, 12 h) stimulated the expression of BNP in both myocytes, while ouabain had no significant effect on BNP expression in either the control or the p85-KO myocytes (Fig. 3b, c).

In separate experiments, we examined the effects of ouabain, ET-1, and the combination of the two on protein synthesis in control cardiomyocytes. The results (Fig. 3d) are consistent with the proposition that ouabain-induced hypertrophy antagonizes ET-1-induced hypertrophy at the cellular level.

### Assessing the prophylactic effect of ouabain on TAC-induced cardiac hypertrophy and dysfunction in normal mice and those deficient in PI3Kα

Supported by the above findings at the cellular level, the aims of the following in vivo experiments were to determine if treatment of mice with low doses of ouabain could prevent or delay the known TAC-induced cardiac dysfunction and failure; and if so, whether or not such ouabain effects were dependent on PI3Kα.

TAC - caused chronic changes were monitored over a period of 8 weeks. To assess ouabain effects, the drug was infused subcutaneously (50 μg/kg/day) by ALZET osmotic pump for continuous dosing during the first 4 weeks. In these experiments, ouabain concentration in circulating blood in heart is dependent on its absorption, distribution, metabolism and excretion. We chose the indicated dosage regimen based on previous finding of others [21] and our data of Fig. 3a in order to keep serum concentrations of ouabain at low nM range (see Discussion).

Because of previous disagreements on whether or not ouabain affects blood pressure in rodents [21, 22], it was necessary to determine the effect of long-term infusion of ouabain on systemic blood pressure in our studies. Tail-cuff blood pressure measurements showed no significant ouabain effects in sham or TAC groups from the control or the p85-KO mice (Additional file 1: Fig. S1). Evidently, ouabain effects on systemic blood pressure are peculiar to rats but not mice as also noted before by others [21].

**Fig. 4:**
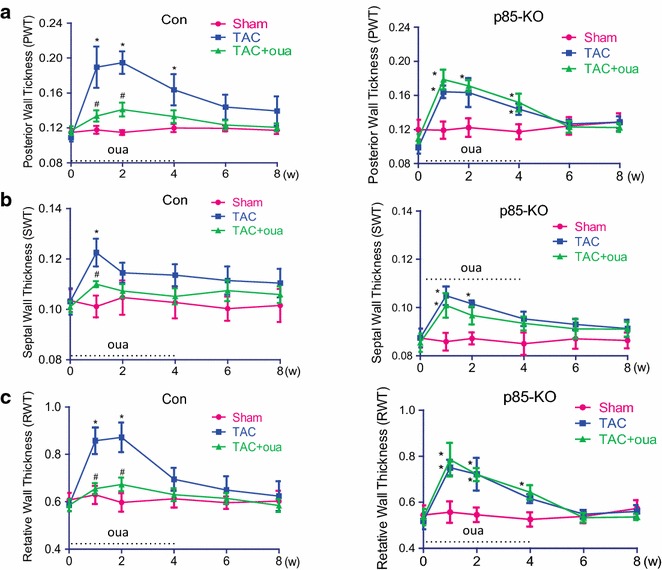
Effects of ouabain on TAC-induced cardiac hypertrophy in Con and p85-KO mice. Experiments were done as described in “Methods”. Left ventricular wall thickness was monitored by echocardiography before and after 8 weeks of the surgery. Ouabain was infused subcutaneously (50 μg/kg/day) by ALZET osmotic pumps for continuous dosing for the first 4 weeks. **a** Posterior wall thickness (PWT); **b** septal wall thickness (SWT) and **c** relative wall thickness (RWT). n = 6 ~ 7, *P < 0.05 vs. Sham;^ #^ *P* < 0.05 vs. TAC

**Fig. 5:**
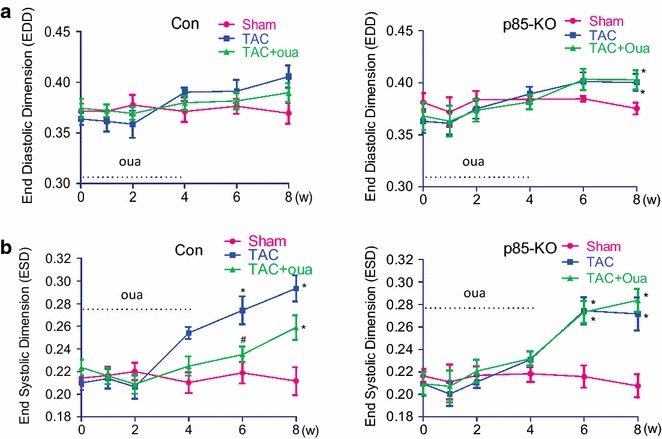
Effects of ouabain on TAC-induced chamber dilation in Con and p85-KO mice. Experiments were done as described in “Methods”. Left ventricular chamber size was monitored by echocardiography before and after 8 weeks of the TAC or sham surgery. Ouabain was infused subcutaneously (50 μg/kg/day) by ALZET osmotic pumps for continuous dosing for the first 4 weeks. **a** End diastolic dimension (EDD); **b** end systolic dimension (ESD). n = 6 ~ 7, *P < 0.05 vs. Sham;^ #^ *P* < 0.05 vs. TAC

**Fig. 6:**
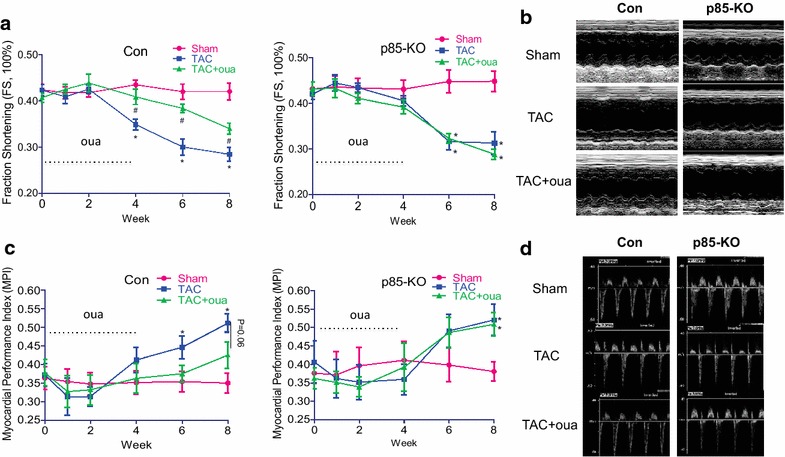
Effects of ouabain on TAC-induced cardiac dysfunction in Con and p85-KO mice. Experiments were done as described in “Methods”. Left ventricular cardiac functions were monitored by echocardiography before and after 8 weeks of the TAC or sham surgery. Ouabain was infused subcutaneously (50 μg/kg/day) by ALZET osmotic pumps for continuous dosing for the first 4 weeks. **a** Fraction shortening, FS % in Con and KO mice. **b** representative echocardiographic M-mode views of mouse left ventricles at week 8 after surgery. **c** myocardial performance index (MPI) in Con and KO mice. **d** representative Pulsed-Wave Doppler flow used for MPI calculation. n = 6 ~ 7, *P < 0.05 vs. Sham;^ #^ *P* < 0.05 vs. TAC

Echocardiographic analysis showed that posterior wall thickness, septal wall thickness and relative wall thickness increased during the first 2 weeks after TAC in the control and the p85-KO mice; however, ouabain prevented the TAC-induced increase of wall thickness in the control but not in the p85-KO mice (Fig. 4). Echocardiography also showed that left ventricular chambers began to dilate after six weeks of TAC in both the control and the p85-KO mice; the effect being especially prominent in the end-systolic dimension (Fig. 5). The early treatment with ouabain during the first 4 weeks significantly reduced this TAC-induced dilation of left ventricles in the control but not the p85-KO mice (Fig. 5). TAC also reduced contractile function (Fractional shortening, FS %) four weeks after surgery in both the control and the p85-KO mice; and ouabain pretreatment rescued cardiac dysfunction in the control but not the KO mice (Fig. 6a, b). Myocardial Performance Index (MPI) generated from pulsed wave Doppler imaging was employed to further examine systolic and diastolic functions. In line with the FS % results, the early treatment with ouabain partially improved cardiac function in the control but not in the p85-KO mice (Fig. 6c, d).

**Fig. 7:**
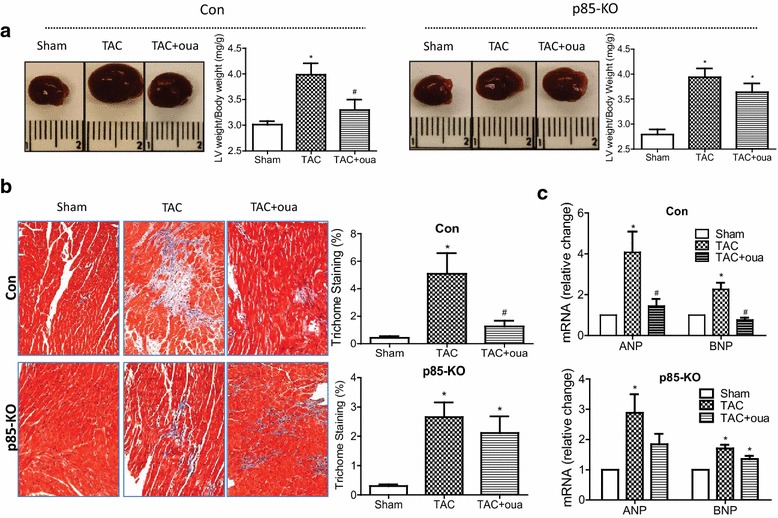
Effects of ouabain on TAC-induced heart weight, cardiac fibrosis and pathological hypertrophic markers after 8 weeks of surgery. **a** Representative heart images (*left*) and quantitative heart weights (*right*); **b** representative images (*left*) and quantitative data (*right*) on cardiac fibrosis by trichrome staining (n = 5) and **c** fetal genes ANP, BNP mRNA expression in Con and p85-KO hearts. n = 6 ~ 7, *P < 0.05 vs. Sham;^ #^ *P* < 0.05 vs. TAC

Postmortem analyses performed after 8 weeks of TAC showed that: (1) Ouabain pretreatment significantly attenuated the TAC-induced cardiac hypertrophy in the control mice but not the KO mice (Fig. 7a); (2) cardiac fibrosis measured by trichrome staining, which was evident in all TAC hearts was significantly reduced by ouabain pretreatment in the control but not the p85-KO hearts (Fig. 7b). We also measured peri-vascular fibrosis in all groups and observed results similar to those of Fig. 7b (data not shown); (3) the TAC-induced effects on ANP and BNP mRNA expressions noted in both the control and the KO mice were antagonized by ouabain pretreatment in the control but not the p85-KO hearts (Fig. 7c).

Taken together, the above in vivo findings provide strong support for the existence of a prophylactic effect of ouabain on TAC-induced cardiac hypertrophy and the subsequent cardiac dysfunction; and indicate that this ouabain effect is dependent on the presence of PI3Kα in the heart.

**Fig. 8:**
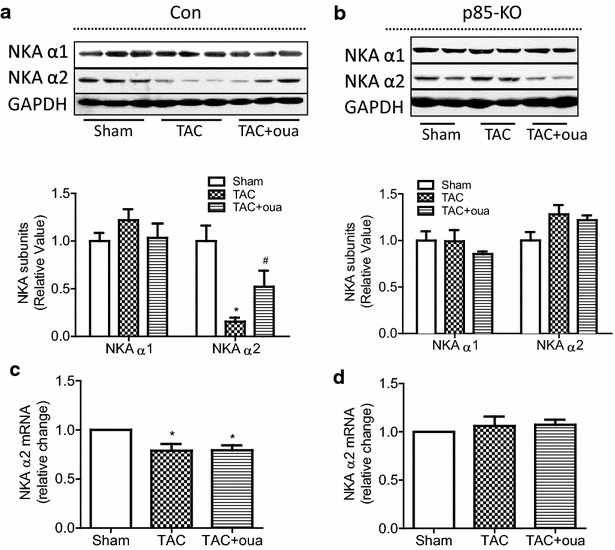
Effects of ouabain on TAC-induced changes on Na^+^/K^+^-ATPase (NKA) isoforms in Con and KO hearts after 8 weeks of surgery. **a** Representative blots (*top*) and quantitative data (*bottom*) of protein expression of NKA α1 and α2 in Con hearts; **b** representative blots (*top*) and quantitative data (*bottom*) of protein expression of NKA α1 and α2 in p85-KO hearts; **c** mRNA expression of NKA α2 in Con hearts and **d** mRNA expression of NKA α2 in KO hearts. n = 6 ~ 7, *P < 0.05 vs. Sham, #*P* < 0.05 vs. TAC

Because of the longstanding prior findings indicating that the α2-subunit of the rat cardiac Na^+^/K^+^-ATPase, but not that of the α1-subunit, may be transcriptionally down-regulated by pressure overload [23, 24], we also looked for changes in the mRNA and protein levels of the α-subunits in the postmortem hearts of the experiments of Fig. 7. In the control mouse hearts, TAC significantly reduced α2 mRNA and protein levels, but did not alter the protein level of the α1-subunit (Fig. 8a, c). In the control mouse treatment with ouabain for the first 4 weeks, the TAC effect on the protein level of the α2 was clearly antagonized by ouabain, though a significant ouabain effect on the α2 mRNA was not detected (Fig. 8a, c). Consistent with the lack of effect of TAC on the expression of the α1-subunit, ouabain treatment also did not affect the expression of α1 protein in the control hearts (Fig. 8a). In the p85-KO mouse hearts, neither TAC nor TAC with ouabain treatment had significant effects on α2 mRNA and α1 and α2 protein levels relative to those of sham-operated mice (Fig. 8b, d).

Taken together, the findings of Fig. 8 clearly indicate the previously unrecognized fact that PI3Kα is required for the downregulation of the α2 isoform of cardiac Na^+^/K^+^-ATPase. Though previous studies [23, 24] showed that TAC effects on α2 are transcriptional, we showed the possibility that ouabain may prevent degradation or alter the translational efficiency of α2 isoform of cardiac Na^+^/K^+^-ATPase.

## Discussion

The decisive part of these studies that establish the prophylactic effect of ouabain on pressure overload-induced hypertrophy are the results of the in vivo experiments on the hearts of the control mice and those deficient in PI3Kα. These studies, however, would not have been attempted had we not done our initial experiments on the isolated cultured myocytes. It is necessary, therefore, to begin with the discussion of the important aspects of the in vitro studies.

### Selective activation of PI3Kα by ouabain and its effects on the hypertrophy of cardiomyocytes

At the cellular level, cardiac hypertrophy is accompanied by the increased size of the cardiac myocytes most of which have little or no capacity to proliferate [25]. In our previous studies on isolated mouse myocytes [26], we showed that the hypertrophy of these cells as measured by increase in protein synthesis is stimulated by ouabain through the activation of PI3Kα/Akt cascade, but we had no direct evidence that the ouabain-activated PI3K was the PI3Kα. Our present findings now demonstrate that only PI3Kα but not PI3Kγ is activated by ouabain (Fig. 2b); and that only in control myocytes but not those deficient in PI3Kα, there is ouabain-induced activation of Akt resulting in hypertrophy (Figs. 2c, d, 3c).

Our ability to study stimulus-induced hypertrophy in isolated adult cardiomyocytes of mice allowed us to ask if the ouabain-induced selective activation of PI3Kα could antagonize cellular hypertrophy induced by a selective activator of PI3Kγ such as ET-1. Our experiments showed that even in these in vitro studies ouabain seemed to reduce ET-1-stimulated hypertrophy (Fig. 2f). In spite of the obvious limitation of these in vitro experiments, their results clearly encouraged the conduct of our subsequent in vivo studies.

The most important part of our findings on the cultured myocytes that allowed us to proceed to the in vivo experiments was the data showing the great sensitivity of PI3Kα/Akt pathway to ouabain concentrations as low as 5 nM (Fig. 2e). This clearly suggested the feasibility of the more onerous in vivo studies. Previous studies of others [21] had indicated that the repeated daily administration of 300 μg/kg of ouabain to mice resulted in extracellular levels of 3.3 nM of “ouabain-like” immune-reactive material and no evident toxicity. It had also been shown that in wild-type mouse hearts, the first evidence of positive inotropic effect is noted at about 40 nM ouabain [27]. Based on this information and our data of Fig. 2e, we chose to use the daily dose of 50 μg/kg of ouabain in our in vivo experiments (Figs. 4, 5, 6, 7, 8) to ensure that ouabain regimen is subinotropic and nontoxic. The results of our in vivo experiments proved that we had made the right choice.

In relation to the very high sensitivity of the PI3Kα/Akt pathway to ouabain (Fig. 2e), it is important to emphasize the contrasting low ouabain sensitivity of the pathway that leads to ERK1/2 activation (Fig. 2f). Although our previous studies [11, 13, 14] had shown that in adult cardiac myocytes two parallel cell signaling cascades; i.e., EGFR-Src/Ras/Raf/ERK and PI3Kα/Akt are functionally linked to the ouabain-inhibited Na^+^/K^+^-ATPase, we had not tested a wide range of ouabain concentration on these pathways before. The present findings (Fig. 2e, f) showing about two orders of magnitude of difference in the sensitivities of these cascades in the adult mouse myocytes suggest that each of these cascades is linked to a different ouabain binding site. And since the two known α-subunits of these myocytes do indeed have ouabain sensitivities of about 2–3 orders of magnitude apart [18, 27], it is reasonable to conclude that the EGFR-Src/Ras/Raf/ERK cascade is functionally linked to the insensitive α1-isoform, and that the PI3Kα/Akt is functionally linked to the sensitive α2-isoform. This conclusion also fits the previously observed irrelevance of the EGFR-Src/Ras/Raf/ERK pathway to ouabain-induced hypertrophy of the mouse cardiomyocytes [14].

### Prevention of pressure overload-induced cardiac dysfunction by treatment with a nontoxic dose of ouabain

The major findings of our in vivo studies and their implications are rather straightforward. In both the control mice and those deficient in cardiac PI3 Kα, a standard protocol of TAC produced compensated hypertrophy during the first 4 weeks, followed by decompensated hypertrophy and failure during the next 4 weeks; and ouabain treatment during the first 4 weeks clearly antagonized TAC-induced cardiac dysfunction in control mice but not in those deficient in cardiac PI3Kα (Figs. 4, 5, 6, 7). In short, our in vivo studies agreed with the findings of Williams and Braunwald [2] regarding the prophylactic effect of digitalis on the hypertrophy of the pressure overloaded heart. The major difference between the two studies done nearly half century apart is that they used rats and digitoxin but we have used mice and ouabain.

Our studies provide a wealth of new information on the mechanistic bases of the above prophylactic effect of ouabain. The most important being that the absence of this prophylactic effect in hearts deficient in PI3 Kα establishes the hypothesis that it is the selective activation of this lipid kinase isoform that opposes the detrimental effects of the various cell signaling cascades that are activated by pressure overload on the heart [13, 15].

**Fig. 9:**
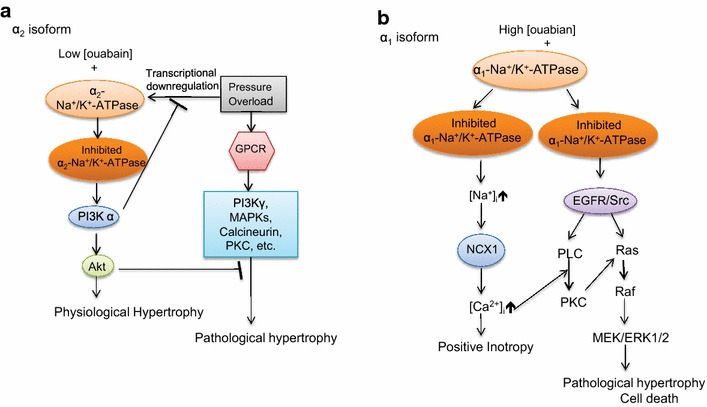
Schematic presentation of ouabain’s different growth-related effects on α1 and α2 isoforms of Na^+^/K^+^-ATPase in the mouse cardiomyocytes. **a** α2-isoform: activation by sub-inotropic ouabain concentrations (< 40 nM) of the signaling cascades that lead to physiological hypertrophy, and block TAC-induced pathological hypertrophy and **b** α1-isoform: induction of positive inotropy by higher concentrations of ouabain, and activation of signaling cascades that leads to pathological hypertrophy and cell death. See “Discussion”

Another important mechanistic issue revealed by our findings is the identification of the α2 isoform of Na^+^/K^+^-ATPase as the likely partner of the PI3Kα for exerting and regulating ouabain’s prophylactic effect on the overloaded heart. It is the responsiveness of the PI3Kα/Akt pathway to low nM ouabain concentrations that clearly implicates the α2-isoform in a functional interaction with PI3Kα resulting in prevention of the hypertrophy. In addition, since pressure-overload down-regulates the α2 isoform and ouabain reverses this (Fig. 8a, c), it seems that there is a second distinct role of ouabain-inhibited α2-isoform: Saving the α2-isoform from disappearance due to overload-induced transcriptional downregulation. The fact that in hearts deficient in PI3Kα neither the overload-induced downregulation of the α2-isoform, nor the ouabain-induced reversal of this are observed (Fig. 8b, d) clearly indicate that PI3Kα must also be involved in the transcriptional downregulation of the cardiac α2 isoform of Na^+^/K^+^-ATPase. The mechanism of this novel action of PI3Kα remains to be studied. A scheme summarizing the above conclusions is presented in Fig. 9.

It is appropriate to note that other recent evidence has also implicated the cardiac α2 isoform of Na^+^/K^+^-ATPase in regulation of cardiac hypertrophy. There is an extensive history of research suggesting the special role of the digitalis-sensitive but quantitatively minor α2 isoform in the regulation of intracellular Ca^2+^ and Na^+^ of the rodent myocyte [18]; and there is the more recent evidence [28] showing that in transgenic mice with cardiac-specific overexpression of α2 isoform, but not in mice with overexpression of α1 isoform, TAC-induced hypertrophy and remodeling are attenuated. While a thorough examination of the relation of our findings to those of Correll et al. [28] is outside the scope of this Discussion, we suggest that the findings of the two laboratories are supportive of each other in emphasizing the need for further studies on how the α2 isoform regulates TAC-induced pathological hypertrophy on the one hand, and ouabain-induced activation of PI3Kα on the other.

Our results indicating that in the control mouse the prophylactic effect of nontoxic ouabain concentrations on the pressure overloaded heart is due to the drug’s interaction with the α2 isoform of cardiac Na^+^/K^+^-ATPase is also significant when we consider the possible relevance of the present animal studies to the therapeutic use of digitalis in man. Because all cardiac α isoforms of Na^+^/K^+^-ATPase in the human heart have high digitalis sensitivities similar to the α2-isoform of the rodent heart [8], it is reasonable to suspect that low nM blood levels of ouabain or another digitalis drug may also exert similar prophylactic effects in man without disturbing intracellular ion concentration. Whether only the human α2-isoform or all three human cardiac isoforms respond to subinotropic ouabain concentrations through the activation of PI3Kα remains to be investigated.

Regarding the potential relevance of the present findings to the clinical use of digitalis, our findings also suggest the following possibility: Since numerous reexaminations of the Digitalis Investigation Group trial [29, 30] have indicated the beneficial effects of lower serum levels of digoxin (0.64–1.15 nM) resulting from the dosage used in the trial, it may be that such therapeutic benefits were induced through the selective activations of PI3Kα and the signaling cascades that are linked to this lipid kinase rather than by safe levels of digoxin-induced positive inotropy. Though it is most commonly assumed that any beneficial effect of digitalis in the treatment of heart failure is due to the drug’s positive inotropic action, it is appropriate to recall that there has never been a consensus on this issue, especially in studies on man [30–32].

## Conclusion

This study establishes that in normal mice low and safe doses of ouabain, a prototypic digitalis drug, prevent or delay cardiac dysfunction and failure that are caused by pressure overload on the heart. Our findings also show that these ouabain effects are exerted through its binding to the α2-isoform of the mouse cardiac Na^+^/K^+^-ATPase, and the resulting activation of PI3Kα/Akt cell signaling cascades. Also clearly indicated by our results is that the known downregulation of the cardiac α2-isoform by pressure overload is prevented by ouabain-induced activation of PI3Kα. In conjunction with a wealth of available information on the clinical use of digitalis drugs in man, the present findings also suggest the need for further studies on the potential use of these drugs for the prevention of heart failure as advocated nearly a century ago.

## Methods

### Animal

Adult male mice of 2–6 months of age were used for this study. PI3K p85α^loxP/loxP^ p85β^−/−^ mice were gifts from Luo et al. [16]. Striated muscle creatine kinase (mck)-Cre transgenic mice (control mice; also referred to as “Con” mice) were purchased from the Jackson Laboratory (Stock #006475). PI3K p85α muscle-specific knockout p85β global knockout mice (p85α^mKO^ p85β^−/−^ mice, referred to as “p85-KO” mice) were crossbred by PI3K p85α^loxP/loxP^ p85β^−/−^ mice with mck-Cre mice. All mice were housed in pathogen-free conditions and maintained with 12 h dark/light cycle with free access to food and water. Animal care and experiments were done following the guidelines of NIH guide for the care and use of laboratory animals and the Institutional Animal Care and Use Committee of the University of Toledo.

### Adult mouse cardiomyocyte isolation and culture

12–16 week old male mice were used for isolation and culture of cardiomyocytes as previously described [14, 26]. Mice were heparinized (5000 U/Kg) and anesthetized with Ketamine (200 mg/kg b.w.)/Xylazine (10 mg/kg b.w.) via intraperitoneal (i.p.) injection. Approximately, 1.0 million viable rod-shaped cells were yielded from one heart. Cardiomyocytes were seeded to the culture dishes (pre-coated with 10 μg/ml laminin) in Modified Eagle’s Medium, supplemented with 2 mM ATP, 2 mM glutamine, 10 % fetal bovine serum (FBS), 10 mM 2,3-butanedione monoxime (BDM), 100 U/ml penicillin and 100 μg/ml streptomycin for 2 h in a 2 % CO_2_ humidified incubator and then cultured overnight in a medium in which FBS was replaced by 0.1 % Bovine Serum Albumin. This medium was then changed to a fresh one that excluded BDM 30 min before the start of the indicated experiments.

### SDS-PAGE/western blot analysis

This was done as described before [13, 33]. Briefly, protein was extracted from cultured cardiomoycytes or homogenized cardiac ventricular tissue in RIPA buffer. Protein extracts (10-80 µg) were combined with Laemmli loading buffer containing 5 % 2-mercaptoethanol, boiled for 5 min, and size fractionated by sodium dodecyl sulfate polyacrylamide gel electrophoresis (SDS PAGE). When extracts were to be subjected to SDS-PAGE for the detection of Na^+^/K^+^-ATPase subunits, they were incubated at 37 °C for 15–30 min instead of being boiled. Proteins were transferred to Polyvinylidene Difluoride membranes. Membranes were incubated for 1 h with 5 % nonfat dried milk in Tris-Buffered Saline Tween-20 buffer and then incubated overnight at 4 °C with primary antibodies. After incubation, membranes were washed, incubated with peroxidase-conjugated secondary antibody, and analyzed using ECL (Perkin-Elmer Life Sciences). Primary antibodies were from BD Transduction Laboratories: anti-PI3 K p110α, anti-PI3 K p85; Cell Signaling Technology: rabbit anti-phospho 473-Akt, anti-Akt; Developmental Studies Hybridoma Bank, University of Iowa (Iowa City, IA): Na^+^/K^+^-ATPase α1 (α6F); ABR: Na^+^/K^+^-ATPase α2; Millipore: Na^+^/K^+^-ATPase β1. Anti-PI3 K p110γ, GAPDH, secondary antibodies goat anti-rabbit IgG-horseradish peroxidase (HRP), and goat anti-mouse IgG-HRP were purchased from Santa Cruz Biotechnology.

### PI3 K lipid kinase assay

This was conducted as previously described [13, 14, 33]. Briefly, cells were lysed in RIPA buffer with inhibitors. Equal amount of protein in each sample were incubated with either anti-PI3 K p85α antibody (06–195, EMD Millipore), or anti-PI3 K p110γ antibody (sc-7177, Santa Cruz) overnight at 4 °C, followed by incubation with Protein A agarose beads for 3 h at 4 °C. And then, the immune complex was washed four times with buffer (100 mM NaCl, 1 mM Na_3_VO_4_, and 20 mM HEPES, pH 7.5) and resuspended in 40 µl of buffer (180 mM NaCl and 20 mM HEPES, pH 7.5). PI3 K activity in the immunoprecipitates was assayed directly on the beads by a standard procedure with PI 0.6 mg/ml (Avanti Polar Lipids, Alabaster, AL, USA) and [γ-^32^P] ATP (250 μM) used as substrates. The reactions were performed at room temperature and stopped after 10 min by addition of 80 µl of 1 M HCl. The lipids were extracted with 160 µl of chloroform–methanol (1:1), spotted on a thin-layer chromatography plate, and separated with chloroform-acetone-methanol-glacial acetic acid-H_2_O (40:15:13:12:8). The reaction product [γ-^32^P] PI(3)P was separated from the origin on the TLC plate and was exposed to storage phosphor screen. The screen was scanned by Typhoon Trio phosphorimager (GE Healthcare, USA) and the top PI(3)P dots were quantified by Image J software.

### Protein synthesis assay

Protein synthesis was measured using [^3^H]-Leucine or [^3^H]-Phenylalanine incorporation assay as previously described [14, 33]. Protein synthesis assay was used as indication of myocyte hypertrophy [14].

### Real-time reverse transcription polymerase chain reaction (RT-PCR)

This was conducted as previously described [14]. Total RNAs were extracted from cardiomyocytes and heart tissues with RNeasy plus mini kit (Qiagen, #74134) and RNeasy Fibrous Tissue Kit (Qiagen, #74704) respectively according to the manufacturer’s instructions. High-capacity cDNA reverse transcription kit (AB applied Biosystems) was used for RT-PCR. Real-time PCR was performed by the SYBR Green method with an Applied Biosystems 7500 Fast Real-Time PCR System. All samples were run in triplicate. In all cases, glyceraldehyde-3-phosphate dehydrogenase (GAPDH) gene was used for data standardization and normalization. Gene expression levels and fold change comparisons were assessed using the ΔCt (cycle threshold) and ΔΔCt, respectively. Primers (forward and reverse, 5′ → 3′) were ANP: TCG TCT TGG CCT TTT GGC T and TCC AGG TGG TCT AGC AGG TTC T; BNP: AAG TCC TAG CCA GTC TCC AGA and GAG CTG TCT CTG GGC CAT TTC; β-MHC: ATG TGC CGG ACC TTG GAA G and CCT CGG GTT AGC TGA GAG ATC A; NKA α1: ATC TGA GCC CAA ACA CCT GCT AGT and AAG CGT CCT TCA GCT CTT CAT CCA; NKA α2: ACA ACC AGA TCC ATG AGG CTG ACA and TTG AGC AGA GCT GAC TAC GAA GCA; and GAPDH: CAT GGC CTT CCG TGT TCC TA and CCT GCT TCA CCA CCT TCT TGA T.

### TAC surgery and osmotic mini-pump implantation

Microsurgical procedure of pressure overload hypertrophy induced through TAC was performed by modification of previously described procedures [34, 35]. Briefly, 12 week-old male mice were anesthetized with isoflurane 5 % and connected to a rodent ventilator. The chest cavity was entered through sternum above the third rib, and aortic constriction was performed by tying a 6–0 silk suture (SP114, LOOK™ suture) against a 27 gauge needle for Con mice or a 25 gauge needle for KO mice, resulting in similar degrees of hypertrophy in both groups (Additional file 1: Fig. S2). Sham operated mice underwent the same operation except for tying the suture loosely. We noted that aortic constriction by a 27-gauge needle on KO mice caused 100 % mortality but all mice survived in sham group. For pain relief, a subcutaneous injection of buprenorphine (0.05–0.1 mg/kg) was administered for each mouse.

Ouabain-filled osmotic mini-pumps (Alzet, #2004) were implanted underneath the dorsal skin 1 day after the surgery under anesthesia by 2–3 % isoflurane. Ouabain (50 μg/kg/day) was continuously infused for 4 weeks. All mice were euthanized at the end of 8 weeks after surgery. Body weight and heart weight were recorded. One part of ventricular free wall was immediately fixed for histological study; the rest of left ventricles were snap-frozen in liquid nitrogen and stored in −80 °C for further analyses.

Mice were subjected to euthanasia by administration of ketamine (200 mg/kg b.w.) and xylazine (10 mg/kg b.w.) i.p. at the end of the experiment.

### Echocardiography

Left ventricular function and geometry was assessed as previously described [36]. Briefly, mice underwent echocardiographic assessment using an ACUSON Sequoia ™ C512 Ultrasound System (Siemens) with a 15-MHz linear array transducer. The mice were anesthetized with isoflurane (2 %) in 100 % oxygen in an anesthesia chamber. Anesthesia was maintained (0.5–1.5 %) by mask. Anesthetized mice were weighed, de-haired, transferred to a heating pad, and placed in a supine position. The parasternal long axis was used to obtain 2-D and 2-D guided M-mode images for the assessment of LV end systolic and diastolic areas, diameters, wall thicknesses and systolic function. Transmitral Doppler flow was traced in parasternal long axis view for measurement of left ventricular isovolumetric contraction time (IVCT), isovolumetric relaxation time (IVRT) and ejection time (ET). Myocardial Performance Index (MPI), which reflects both cardiac diastolic and systolic function, was calculated by the following equation: MPI = (IVCT + IVRT)/ET.

### Catheterization of mouse left ventricles

The procedure was conducted under a stereomicroscope according to the published protocol [37]. Mice were heparinized (5000 U/kg) and anesthetized with Ketamine (50–150 mg/kg) and Xylazine (2.5–7.5 mg/kg) via intraperitoneal (i.p.) injection. Mice were placed on the surgical platform and kept warm at 37 °C. MPVS UltraTM Pressure–Volume (P–V) system (Millar Instruments, Inc.) and PowerLab 8/30 (ADInstruments, Inc.) were turned on for stabilization 30 min prior to the recording. The mouse Pressure–Volume conductance catheter (SPR-839, Millar Instruments, Inc.) was advanced into the left ventricle through right carotid artery. After stabilization of the signal for 10–15 min, the pressure and volume signals (P–V loops) were acquired and recorded by PowerLab 8/30 (ADInstruments, Inc.) and MPVS UltraTM Pressure–Volume (P–V) system (Millar Instruments, Inc.). Real-time P–V loop and other cardiac functions (e.g. HR, dp/dt, etc.) were monitored and data analysis was conducted with Labchart 7 (ADInstruments, Inc.) software.

### Masson trichrome staining

Left ventricle was fixed in 10 % buffered formalin conducted by the standard Masson’s trichrome staining procedure. The staining was shown as nuclei (black), cytoplasm, muscle fibers (red) and collagen (blue). Images were taken by the Olympus IX51 inverted microscope connected to SPOT Insight 2.0 Mp Camera under a 20 × objective lens. Seven-fourteen pictures were taken from each heart sample. The percentage of blue area in each heart section was quantified by Image J software (National Institutes of Health, USA).

### Data analysis

All data are presented as mean ± standard error. Statistical analyses were performed by using GraphPad Prism 5.0 software (La Jolla, CA, USA). The Student’s t test was used to compare two groups. One-way analysis of variance (ANOVA), followed by the Bonferroni’s post hoc test was used to compare multiple groups. Other method was listed in the figure legend. Differences were considered statistically significant at p < 0.05.

## Additional file

10.1186/s13578-015-0053-7 Effect of ouabain infusion on systemic blood pressure in Con and p85-KO mice. Mice were grouped as sham, sham+oua (50 μg/kg/day b.w.), TAC or TAC+oua (50 μg/kg/day b.w.). Ouabainfilled osmotic mini-pumps (Alzet, #2004) were implanted underneath the dorsal skin one day after the surgery. Ouabain (50 μg/Kg/day) was continuously infused for 4 weeks. All mice were euthanized at the end of 8 weeks after surgery. Blood pressure was measured by tail-cuff volume-pressure recording (VPR) every two weeks. A. Con: sham (n=7), sham+oua (n = 6), TAC (n = 7), TAC+oua (n = 6); B. p85-KO: sham (n = 5), sham+oua (n = 4), TAC (n = 4), TAC+oua (n = 6). **Figure S2.** Comparison of TAC-induced cardiac hypertrophy in Con and p85-KO mice. Experiments were done as described in Methods. Left ventricular wall thickness was monitored by echocardiography before and after eight weeks of the surgery. A. Relative wall thickness (RWT). n = 6~7, * P < 0.05 v.s. Sham; B. Comparison of TAC-induced hypertrophy between Con and p85-KO mice. There is no difference between two groups by repeated measures ANOVA. * P < 0.05.
