# Supplementary material for: Ouabain prevents pathological cardiac hypertrophy and heart failure through activation of phosphoinositide 3-kinase α in mouse
Source: Cell Biosci. 2015 Nov 18;5:64. doi: 10.1186/s13578-015-0053-7 (PMC4652409; doi:10.1186/s13578-015-0053-7)
Supplement: Supplementary file 1 — 10.1186/s13578-015-0053-7 Effect of ouabain infusion on systemic blood pressure in Con and p85-KO mice. Mice were grouped as sham, sham+oua (50 μg/kg/day b.w.), TAC or TAC+oua (50 μg/kg/day b.w.). Ouabainfilled osmotic mini-pumps (Alzet, #2004) were implanted underneath the dorsal skin one day after the surgery. Ouabain (50 μg/Kg/day) was continuously infused for 4 weeks. All mice were euthanized at the end of 8 weeks after surgery. Blood pressure was measured by tail-cuff volume-pressure recording (VPR) every two weeks. A. Con: sham (n=7), sham+oua (n = 6), TAC (n = 7), TAC+oua (n = 6); B. p85-KO: sham (n = 5), sham+oua (n = 4), TAC (n = 4), TAC+oua (n = 6). Figure S2. Comparison of TAC-induced cardiac hypertrophy in Con and p85-KO mice. Experiments were done as described in Methods. Left ventricular wall thickness was monitored by echocardiography before and after eight weeks of the surgery. A. Relative wall thickness (RWT). n = 6~7, * P < 0.05 v.s. Sham; B. Comparison of TAC-induced hypertrophy between Con and p85-KO mice. There is no difference between two groups by repeated measures ANOVA. * P < 0.05. [file 13578_2015_53_MOESM1_ESM.pdf]

Supplementary Figure. 1

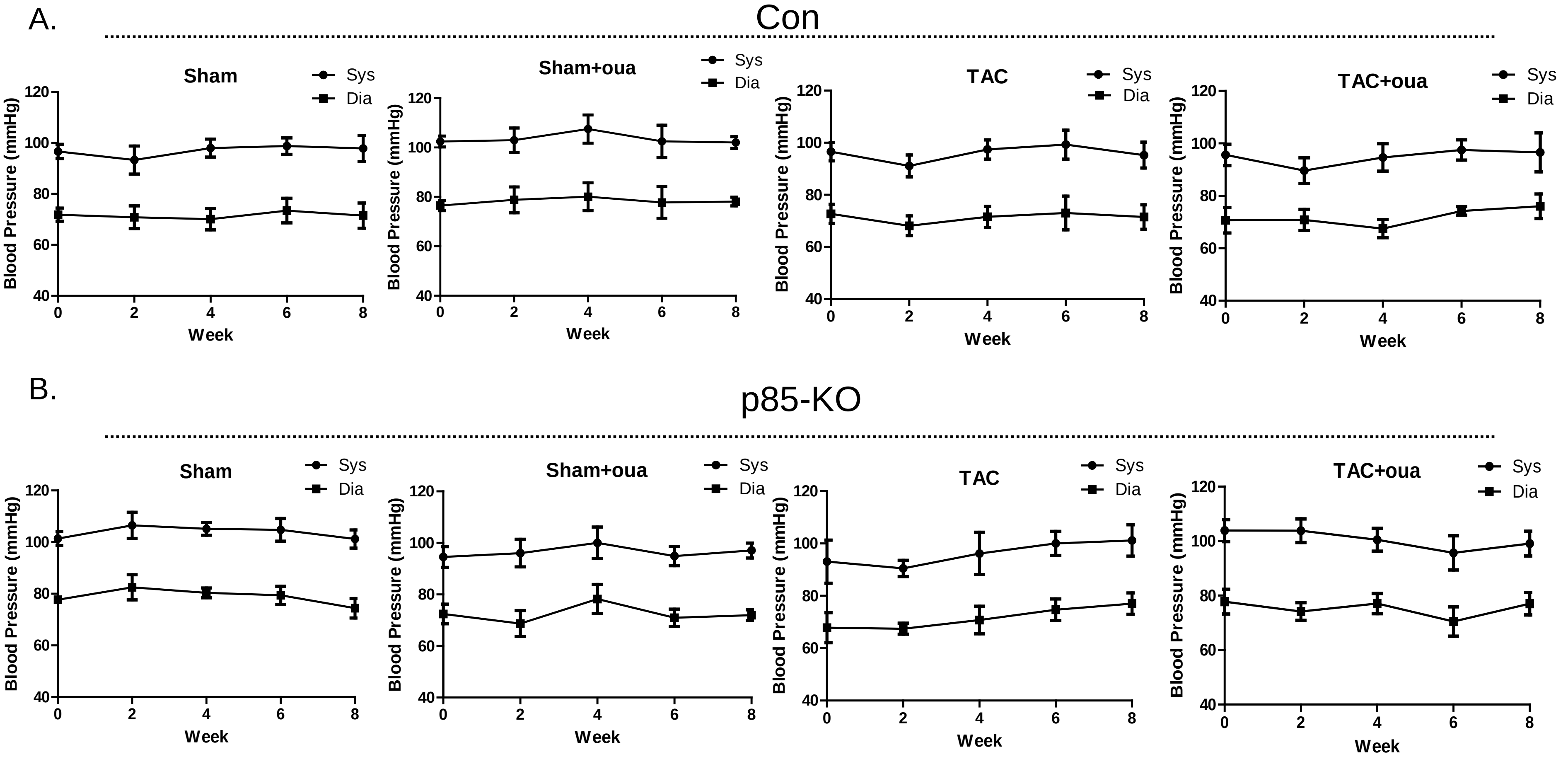

Figure 1. Effect of ouabain infusion on systemic blood pressure in Con and p85-KO mice. Mice were grouped as sham, sham+oua (50μg/kg/day b.w.), TAC or TAC+oua (50μg/kg/day b.w.). Ouabain-filled osmotic mini-pumps (Alzet, #2004) were implanted underneath the dorsal skin one day after the surgery. Ouabain (50μg/Kg/day) was continuously infused for 4 weeks. All mice were euthanized at the end of 8 weeks after surgery. Blood pressure was measured by tail-cuff volume-pressure recording (VPR) every two weeks. A. Con: sham (n=7), sham+oua (n=6), TAC (n=7), TAC+oua (n=6); B. p85-KO: sham (n=5), sham+oua (n=4), TAC (n=4), TAC+oua (n=6).

Supplementary Figure. 2

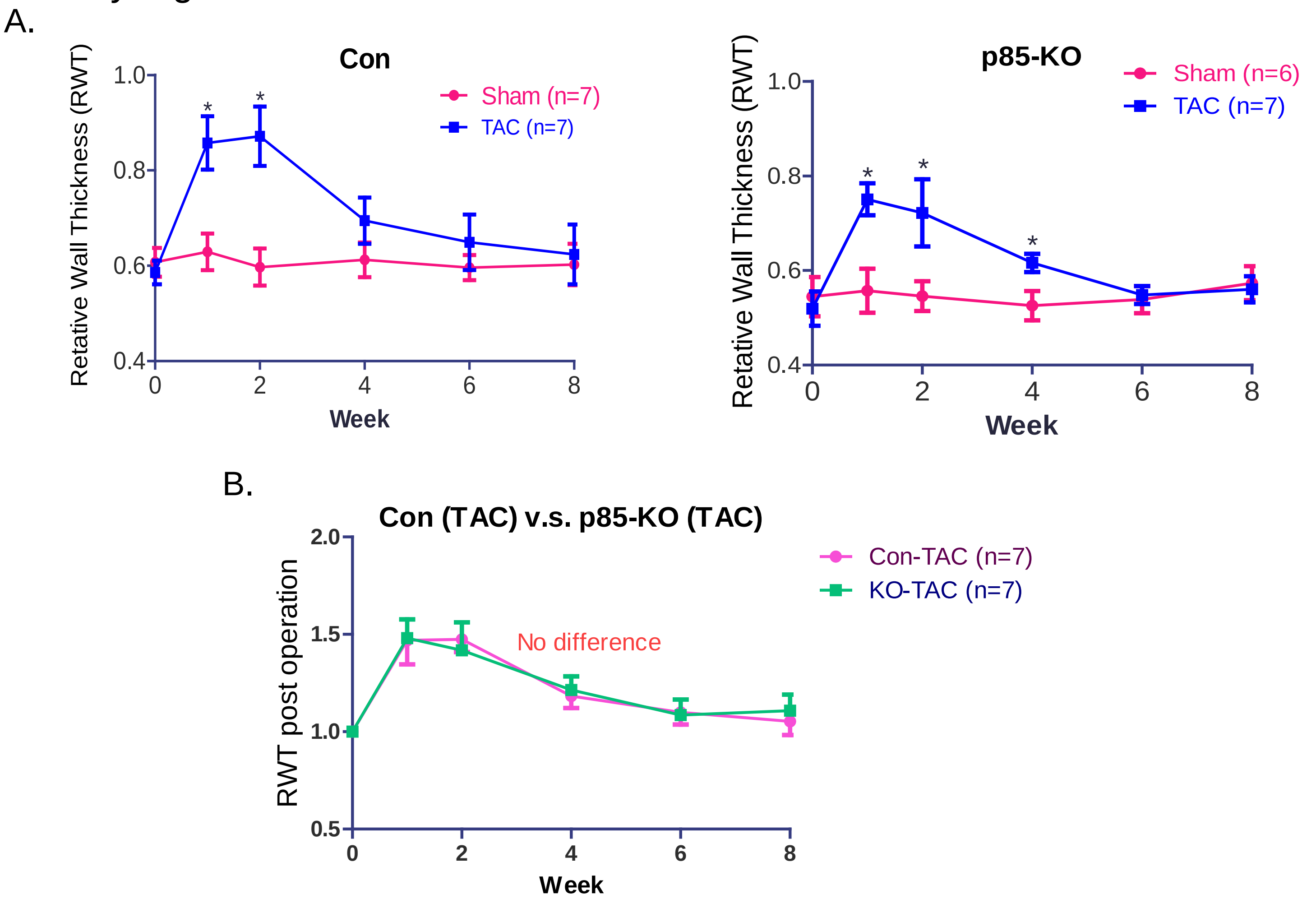

Figure 2. Comparison of TAC-induced cardiac hypertrophy in Con and p85-KO mice. Experiments were done as described in Methods. Left ventricular wall thickness was monitored by echocardiography before and after eight weeks of the surgery. **A.** Relative wall thickness (RWT). n=6~7, * *P*<0.05 *v.s.* Sham; **B**. Comparison of TAC-induced hypertrophy between Con and p85-KO mice. There is no difference between two groups by repeated measures ANOVA. * *P* <0.05.
